# Leistungssteigernde Mittel im Alltag und im Arbeitsleben

**DOI:** 10.1007/s00103-026-04254-2

**Published:** 2026-06-05

**Authors:** Sascha Milin, Felix A. Ebel, Ingo Schäfer

**Affiliations:** 1 Klinik für Psychiatrie und Psychotherapie, Universitätsklinikum Hamburg-Eppendorf, Hamburg, Deutschland; 2 Zentrum für Interdisziplinäre Suchtforschung der Universität Hamburg (ZIS), Universitätsklinikum Hamburg-Eppendorf (UKE), Martinistraße 52, 20246 Hamburg, Deutschland

**Keywords:** Leistungsbezogener Substanzgebrauch, Arbeitsleben, Neuroenhancement, Präsentismus, Public Health, Performance-related substance use, Work environment, Neuroenhancement, Presenteeism, Public health

## Abstract

Der Einsatz potenziell leistungssteigernder Mittel im Alltag und Berufsleben steht häufig im Zusammenhang mit Arbeitsverdichtung, Zeitdruck und begrenzten Erholungsmöglichkeiten. Diese narrative Übersichtsarbeit fasst aktuelle Studien zu Nutzungskontexten, Motiven, Substanzgruppen, Prävalenzen sowie Nutzen und Risiken zusammen.

Die Studien zeigen, dass der Konsum leistungssteigernder Substanzen vor allem kurzfristige Leistungsanforderungen unterstützen soll. Er erfolgt insbesondere bei Müdigkeit, hohen kognitiven Anforderungen, Stress, emotionaler Belastung sowie körperlichen Beschwerden beim Arbeiten u. a. im Zusammenhang mit Präsentismus. Verwendet werden Stimulanzien, Mittel zur Stimmungs- oder Schlafregulation, Schmerzmittel und Nahrungsergänzungsmittel. Bis auf Letztere sind die Substanzen häufig verschreibungspflichtig; einige Stimulanzien sind illegal. Die Prävalenzwerte unterscheiden sich stark aufgrund unterschiedlicher Studienmethoden, Settings und Definitionen für leistungssteigernde Mittel. Allgemein akzeptierte Substanzen werden häufiger genutzt.

Der Nutzen der Substanzen wird meist als begrenzt und kontextabhängig beschrieben. Zentrale Risiken umfassen Toleranzentwicklung, Abhängigkeit, Residualeffekte und sicherheitsrelevante Beeinträchtigungen. Kurzfristige Vorteile stehen oft langfristigen gesundheitlichen und funktionalen Kosten gegenüber. Im Kontext von Public Health und Arbeitsmedizin kann leistungsbezogener Substanzgebrauch auf strukturelle Belastungen hinweisen. Prävention, Versorgung und Arbeitsgestaltung sollten darauf abzielen, die Funktionsfähigkeit nachhaltig zu sichern.

## Abstract (en)

The use of potential performance-enhancing substances in everyday and working life is often linked to an increased workload, time pressure and limited opportunities for rest. This narrative review summarises recent studies on contexts of use, motives, substance groups, prevalence rates, and benefits and risks.

The studies show that the use of performance-enhancing substances is primarily intended to support short-term performance demands. It occurs in particular in cases of fatigue, high cognitive demands, stress, emotional strain and physical complaints at work, including in connection with presenteeism. The substances used include stimulants, mood- or sleep-regulating agents, painkillers and dietary supplements. With the exception of the latter, these substances are often prescription-only; some stimulants are illegal. Prevalence figures vary widely due to differing study methods, settings and definitions of performance-enhancing substances. Generally accepted substances are used more frequently.

The benefits of these substances are generally described as limited and context dependent. Key risks include the development of tolerance, dependence, residual effects and impairments affecting safety. Short-term benefits are often offset by long-term health and functional costs. In the context of public health and occupational medicine, performance-related substance use may indicate structural stressors. Prevention, care and work design should aim to ensure long-term functional capacity.

## Einleitung

Der Gebrauch potenziell leistungssteigernder Mittel ist im Alltag und Arbeitsleben kein Randphänomen. Im Folgenden verstehen wir unter „leistungsbezogenem Gebrauch“ den Versuch, Funktionsbereiche wie Wachheit, Konzentration oder Belastbarkeit kurzfristig zu verändern, um Anforderungen in Arbeit, Ausbildung oder Alltag besser zu bewältigen. Dies umfasst sowohl eine Steigerung über das individuell Übliche hinaus als auch die Aufrechterhaltung von Leistungsfähigkeit unter Belastung [1–3]. Der Begriff wird dabei in einem erweiterten Sinne verwendet und schließt neben kognitiven auch affektive und körperliche Funktionsbereiche ein. Zu beachten ist, das potenziell leistungssteigernde Mittel nicht zwangsläufig zu messbaren Leistungssteigerungen führen und gleichzeitig andere Bereiche wie Erholung, Urteilsqualität oder Gesundheit beeinträchtigen können [4–6].

In vielen Lebensbereichen steigen die Anforderungen an kognitive Verfügbarkeit, emotionale Stabilität und flexible Anpassung. Mehrfachbelastungen, etwa durch die Kombination von Erwerbsarbeit, Sorgearbeit und Ausbildung, führen zu verkürzten Erholungsphasen und häufigeren Belastungsspitzen [7, 8]. Vor diesem Hintergrund gewinnen Strategien an Bedeutung, die eine kurzfristige Optimierung der Funktionsfähigkeit versprechen. Einige der selbstberichteten Konsummotive werfen dabei die Frage auf, ob die vermeintliche Optimierung nicht auch als Bewältigungsstrategie bei bislang nicht diagnostizierten psychischen Beeinträchtigungen oder Belastungsfolgen verstanden werden kann [9].

Aus einer Public-Health-Perspektive sind neben potenziellen gesundheitlichen Risiken auch Auswirkungen auf Sicherheit und Fehleranfälligkeit bei bestimmten Tätigkeiten relevant. Darüber hinaus stellen sich Fragen zu sozialen Normen und zur Verfügbarkeit entsprechender Substanzen, etwa im Hinblick auf rezeptpflichtige versus frei verkäufliche Präparate, Online-Märkte oder die Weitergabe im sozialen Umfeld. Die Debatte bewegt sich somit im Spannungsfeld zwischen Nutzenannahmen, Risiko- und Autonomiefragen sowie der Verantwortung von Organisationen und Versorgungssystemen [10].

Sportdoping und der Gebrauch potenziell leistungssteigernder Mittel im Alltag und Arbeitsleben folgen ähnlichen Zielen, unterscheiden sich jedoch deutlich in ihren Norm- und Regulierungsrahmen. Leistungsbezogener Substanzgebrauch kann – etwa im Studierendenkontext – individuell als funktionale Bewältigungsstrategie verstanden werden, wird jedoch in Befragungsstudien und experimentellen Arbeiten mehrheitlich kritisch bewertet, insbesondere im Hinblick auf Fragen der Fairness [11, 12]. Zugleich zeigen Studien, dass Nutzende den Konsum häufig situationsbezogen begründen, etwa zur Aufrechterhaltung von Leistungsfähigkeit oder zur Bewältigung von Belastungen, und ihn teilweise als Form der Selbstbehandlung einordnen. Insgesamt ergibt sich eine heterogene Bewertung: Während ein Teil den Gebrauch ablehnt, wird er unter bestimmten Bedingungen von einem relevanten Anteil als akzeptabel eingeschätzt [2, 9]. Erkenntnisse zu tatsächlichen Leistungsgewinnen sind begrenzt und uneinheitlich, wie auch systematische Übersichtsarbeiten und Metaanalysen zeigen.

In Bezug auf das Sportdoping besteht ein formalisiertes System aus Verboten, Kontrollen und Sanktionen (Tab. 1). Die Welt-Anti-Doping-Agentur (WADA) definiert Doping unter anderem anhand potenzieller Leistungssteigerung, Gesundheitsgefährdung und des Verstoßes gegen den Geist des Sports, womit es als Regelbruch und Betrug im Wettbewerb gilt [13]. Im Alltag und in der Arbeitswelt fehlt ein vergleichbares Kontrollregime: Substanzgebrauch bleibt überwiegend privat und wird lediglich durch das Arznei- und Betäubungsmittelrecht sowie arbeits- und sicherheitsrelevante Vorgaben berührt. Analog zur Unterscheidung zwischen Doping und legaler Leistungssteigerung wird insbesondere im Bereich kognitiver Funktionen – einschließlich Aufmerksamkeit, Wachheit und Entscheidungsfähigkeit – zwischen nichtmedizinischem Gebrauch verschreibungspflichtiger oder illegaler Substanzen und der Nutzung frei verfügbarer Präparate differenziert [14]. In Alltags- und Arbeitskontexten verschwimmen diese Grenzen häufig, da beide Formen ohne medizinische Indikation eingesetzt werden [10, 15]. Auch Ziele und Motive unterscheiden sich: Während im Sport die wettkampfrelevante Leistungssteigerung im Vordergrund steht, zielt leistungsbezogener Substanzgebrauch im Alltag und Arbeitsleben häufiger auf Teilfunktionen wie Aufmerksamkeit, Wachheit oder Entscheidungsfähigkeit [3, 16, 17]. Solche Verbesserungen können mit Einbußen in anderen Bereichen einhergehen, sodass eine Steigerung der Gesamtleistung nicht zwangsläufig erfolgt [18].

**Tab. 1:** Zentrale Unterschiede zwischen Sportdoping und Leistungssteigerung in Alltag und Arbeitswelt

	Sportdoping	Alltag/Arbeitswelt
Kontrollsystem	Institutioneller und rechtlicher Rahmen (Verbote, Kontrollen, Sanktionen)	Meist privater Substanzgebrauch, lediglich berührt durch Arzneimittel‑/Betäubungsmittel-Recht sowie Vorgaben zur Arbeitssicherheit
Ziele und Motive	Wettkampfbezogen	Situations- und bewältigungsorientiert (z. B. Müdigkeit, Fokus, Stress, Schlafsteuerung)
Unterscheidung zwischen nichtmedizinischem Gebrauch verschreibungspflichtiger oder illegaler Substanzen und frei verfügbaren Präparaten	Klarere Unterscheidung	Oft unklare Unterscheidung, Medikamente werden außerhalb spezifischer Indikationen genutzt

Während Doping im Sport in einem klar definierten institutionellen und rechtlichen Rahmen reguliert ist, unterliegt leistungsbezogener Substanzgebrauch im Alltag und Arbeitsleben nur normativen und teilweise regulatorischen Vorgaben, die je nach Art der Substanz unterschiedlich geregelt und bewertet werden. Stimulanzien etwa werden strenger reguliert als apothekenpflichtige Analgetika.

Das Ziel dieser narrativen Übersichtsarbeit ist, den Gebrauch potenziell leistungssteigernder Mittel im Alltag und Arbeitsleben einzuordnen und praxisnahe Schlussfolgerungen abzuleiten. Zentrale Bezugspunkte sind der Gesundheitsreport der Deutschen Angestellten-Krankenkasse (DAK) zum Themenfeld Doping am Arbeitsplatz [19] und das DFG-Projekt ENHANCE^1^, eine bevölkerungsrepräsentative Panelstudie zu Cognitive Enhancement in Deutschland, sowie die darauf basierenden Veröffentlichungen. Im Fokus steht zunächst, mit welchen Anwendungsmotiven potenziell leistungssteigernde Mittel genutzt werden und wie diese Nutzung beschrieben wird. Dabei werden auch strukturelle Rahmenbedingungen wie Arbeitsverdichtung, Normen und Erreichbarkeit berücksichtigt. Anschließend werden die zentralen Substanzgruppen systematisch beschrieben. Für diese Kontexte wird auf Prävalenzen, Konsummuster bzw. -motive, Zugangswege und gesundheitliche Risiken entlang der aktuellen Literatur eingegangen.

Methodisch folgt der Beitrag einem narrativen Übersichtsansatz mit iterativer Literaturrecherche. Die Auswahl der Anwendungskontexte orientiert sich an der Literaturrecherche und den Fragestellungen zur Leistungssteigerung im Alltags- und Berufskontext in einem weit gefassten Sinne. Die genannten Bereiche sind nicht abschließend; aus Gründen der Fokussierung wurden beispielsweise sexuelle Leistungssteigerung sowie Substanzen wie Alkohol und Nikotin ausgeschlossen.

## Konsummotive im Alltag und Arbeitsleben

### Unterstützung von Wachheit und Kognition

Der Gebrauch potenziell leistungssteigernder Mittel zur Unterstützung von Wachheit und kognitiver Leistungsfähigkeit erfolgt vor allem in Situationen anhaltender Müdigkeit, hoher kognitiver Anforderungen oder enger zeitlicher Vorgaben. Kognition beschreibt dabei mentale Fähigkeiten und Prozesse im Zusammenhang mit Verarbeitung und Wiedergabe von Informationen sowie Wissen und umfasst unter anderem Aspekte der Aufmerksamkeit, Wahrnehmung, Psychomotorik, Erinnerung und exekutiver Funktionen [20]. Typische Anwendungskontexte sind Schicht- und Nachtarbeit, lange Arbeitszeiten, hohe Arbeitsverdichtung sowie Prüfungs- und Lernphasen. Auch Mehrfachbelastungen, etwa durch parallele Erwerbs- und Sorgearbeit, begünstigen den Einsatz [21]. Empirische Studien zeigen, dass der Gebrauch stark vom Setting, der Definition und der betrachteten Substanzgruppe abhängt. Für verschreibungspflichtige oder illegale Substanzen zur kognitiven Leistungssteigerung werden in Deutschland überwiegend 1‑Jahres-Prävalenzen im einstelligen Prozentbereich berichtet, während die Lebenszeitprävalenzen höher liegen können. In Studierendenstichproben werden für den nichtmedizinischen Gebrauch verschreibungspflichtiger Medikamente zur Leistungssteigerung 12-Monats-Prävalenzen im einstelligen bis niedrig zweistelligen Bereich berichtet [22, 23]. In berufstätigen Populationen liegen entsprechende Angaben in der Regel im unteren einstelligen Bereich [21, 24, 25]. Werden frei verfügbare „Soft-Enhancer“ wie koffeinhaltige Getränke oder Nahrungsergänzungsmittel einbezogen, steigen die Prävalenzen deutlich an und erreichen teils Werte von über 60 %, was die starke Definitionsabhängigkeit der Befunde verdeutlicht [24, 26].

Das eingesetzte Substanzspektrum reicht von verschreibungspflichtigen Stimulanzien wie Methylphenidat oder Modafinil über frei verfügbare Produkte wie Koffein und Energy-Drinks bis hin zu illegalen Stimulanzien, die insgesamt selten sind, aber punktuell in Hochbelastungssituationen eingesetzt werden [26]. Diese Substanzen können mit unerwünschten Nebenwirkungen einhergehen wie Insomnie, Kopfschmerzen oder Magenbeschwerden [26]. Insbesondere bei verschreibungspflichtigen und illegalen Stimulanzien kann es auch zu schweren und lebensgefährlichen Nebenwirkungen kommen [10].

### Affekt- und Stressregulation

Der leistungsbezogene Substanzgebrauch zur Affekt- und Stressregulation stellt im Alltag und Arbeitsleben einen weiteren Anwendungsschwerpunkt dar. Im Vordergrund steht die Regulation von Angst, Anspannung oder emotionaler Erschöpfung, um Anforderungen unter Zeit‑, Verantwortungs- oder Erwartungsdruck zu bewältigen [27, 28]. Diese Funktionen sind nicht immer trennscharf von wachheits- und kognitionsbezogenem Gebrauch (siehe oben) abzugrenzen, da Substanzen zur Unterstützung von Wachheit, Antrieb oder Konzentration zugleich zur Bewältigung von Stress- und Belastungserleben eingesetzt werden können. Empirische Arbeiten zeigen konsistent, dass wahrgenommener Stress ein zentraler Prädiktor für den Einsatz solcher Mittel ist, während Resilienzfaktoren protektiv wirken [29].

In der Literatur wird zwischen bewältigungs- und optimierungsorientiertem Gebrauch unterschieden. *Bewältigungsorientierter Gebrauch* zielt auf Stressabbau, Angstreduktion oder Stimmungsstabilisierung ab und wird meist als reaktive Kompensationsstrategie bei Überforderung oder Erschöpfung beschrieben. Demgegenüber bezeichnet *optimierungsorientierter Gebrauch* den proaktiven Einsatz von Substanzen, um unter hohem Leistungs- und Erwartungsdruck die Funktionsfähigkeit oder emotionale Kontrolle aufrechtzuerhalten oder zu steigern.

Studien im Arbeitskontext verknüpfen insbesondere „Overcommitment“ (ein starkes persönliches Engagement, das zu Überlastung führt), ein Ungleichgewicht zwischen Aufwand und Belohnung sowie hohe emotionale Anforderungen mit einem erhöhten Risiko für den nichtmedizinischen Gebrauch verschreibungspflichtiger Medikamente [25, 30]. Dieses Muster lässt sich als *instrumenteller Gebrauch* beschreiben, bei dem Substanzen gezielt zur kurzfristigen Aufrechterhaltung oder Steuerung von Funktionsfähigkeit eingesetzt werden, hier zur Bewältigung von Stress und Leistungsanforderungen. Diese Befunde verweisen auf die Bedeutung der strukturellen Arbeitsbedingungen. Die Abgrenzung zwischen Therapie, Selbstmedikation und Enhancement ist in diesem Bereich besonders relevant.

Verschreibungspflichtige Medikamente werden auch *affektbezogen* gebraucht, also zur Linderung subjektiver psychischer Belastungen. Dies erfolgt häufig ohne formale Diagnose und ärztliche Begleitung sowie außerhalb der jeweiligen Indikation [29, 31]. Dadurch verschwimmen die Grenzen zwischen Behandlung und leistungsbezogenem Gebrauch. Besonders ausgeprägt ist der affekt- und stressbezogene Einsatz in Kontexten mit hohem Leistungs- und Verantwortungsdruck, etwa in medizinischen Berufen oder verschiedenen anderen Berufsgruppen mit besonderen emotionalen Anforderungen [21, 32].

Zum Einsatz kommen verschiedene Substanzgruppen, darunter Anxiolytika, Betablocker, schlafanstoßende Substanzen, teils in Kombination mit frei verfügbaren Substanzen wie Koffein [33]*.* Mit diesem Gebrauch sind spezifische Risiken verbunden, darunter Rebound-Effekte (verstärkte Rückkehr der Symptome, die Anlass für den Gebrauch der Substanz waren, nach Abklingen der Wirkung), gesundheitliche Folgen von Mischkonsum sowie Abhängigkeits- und Toleranzentwicklung, insbesondere bei wiederholtem Gebrauch ohne medizinische Begleitung [34–36]. Auch in Bezug auf diese Funktionalität zeigt sich die enge Verknüpfung mit strukturellen Bedingungen von Arbeit, Ausbildung und Alltag [24].

### Steuerung von körperlicher Belastbarkeit und Schmerz

Viele Formen des leistungsbezogenen Substanzgebrauchs im Alltag folgen weniger einer Optimierungs- als einer Funktionalitätslogik: Ziel ist es, trotz Schmerzen, Infekt oder körperlicher Überlastung handlungs- und arbeitsfähig zu bleiben. Diese Logik entspricht dem Konzept des „Präsentismus“ und beschreibt das Weiterarbeiten trotz gesundheitlicher Beschwerden, etwa bei körperlich belastenden Tätigkeiten, Schichtarbeit, Zeitdruck oder paralleler Erwerbs- und Sorgearbeit, wobei Symptome kurzfristig gedämpft werden [37, 38].

Für diesen Bereich liegen vergleichsweise gute Prävalenzdaten vor, die jedoch stark von Definition, Zeitraum und Setting abhängen. Internationale Studien berichten hohe Nutzungsraten insbesondere für Analgetika und Antiphlogistika im Arbeitskontext, während verschreibungspflichtige Substanzen wie Opioide vor allem in bestimmten Berufsgruppen oder anlassbezogen sichtbar werden [39, 40]. Dies unterstreicht die breite Bedeutung frei verfügbarer Schmerz- und Erkältungsmittel im Arbeitsalltag.

Am häufigsten eingesetzt werden nichtopioide Analgetika und Antiphlogistika, insbesondere nichtsteroidale Antirheumatika (NSAR) und Paracetamol, häufig auch in Kombinations- und Erkältungspräparaten. Opioide spielen seltener, etwa nach Verletzungen, Operationen oder bei chronischen muskuloskelettalen Schmerzen, eine Rolle, sind dann jedoch häufig eng mit Fragen der Arbeitsfähigkeit und des „Durchhaltens“ verknüpft [40].

Die Risiken betreffen sowohl Nebenwirkungen als auch eine mögliche Symptommaskierung. Für NSAR sind gastrointestinale, renale und kardiovaskuläre Risiken relevant, während bei Opioiden Sedierung, Abhängigkeits- und Toleranzentwicklung sowie ungünstige arbeitsbezogene Effekte im Vordergrund stehen. Untersuchungen zeigen – wie oben dargestellt –, dass dies mit längeren Fehlzeiten und geringeren Rückkehrquoten an den Arbeitsplatz verbunden ist [41].

Im vorliegenden Artikel werden ärztlich verordnete Schmerzmittel einbezogen, da bei Berufen mit körperlicher Arbeit vor dem Hintergrund der Präsentismus-Debatte hinterfragt werden sollte, ob schmerzlindernde Mittel auch mit ärztlichem Wissen eingesetzt werden, um krankheitsbedingte Ausfälle zu vermeiden.

## Prävalenz des Gebrauchs leistungssteigernder Mittel

Empirische Studien zum Gebrauch potenziell leistungssteigernder Mittel im Alltag und Arbeitsleben zeigen stark heterogene Prävalenzen, die vom Anwendungskontext [21, 24] sowie von den Eigenschaften der Nutzerinnen und Nutzer [42] abhängen. Unterschiede ergeben sich insbesondere aus Stichprobencharakteristika, Setting und Operationalisierung der Items, sodass sich die Befunde eher anhand wiederkehrender Muster als einzelner Prozentwerte einordnen lassen. Tab. 2 gibt einen Überblick über Prävalenzangaben aus Studien, die verschiedene Settings und Substanzgruppen untersucht haben. Aufgrund der methodischen Heterogenität sind statistische Vergleiche oder Synthesen jedoch nicht möglich. Die exemplarisch ausgewählten Studien unterscheiden sich teils deutlich in Design, Methodik und Stichprobenmerkmalen; die Rücklaufquoten reichen von 13,9 % [23] bis 57,3 % [24, 25].

**Tab. 2:** Ausgewählte Prävalenzstudien zum leistungsbezogenen Substanzgebrauch im Alltag und Arbeitsleben – 12-Monats- und Lebenszeitprävalenz

Studie (Jahr, Land)	Design und Stichprobe	Funktionskontext	Substanzgruppen	Prävalenz	
*Allgemeinbevölkerung*				*12-Monats-Prävalenz*	*Lebenszeitprävalenz*
Bagusat et al. (2018, DE; [29])	Repräsentative Face-to-Face-Befragung Erwachsener (*n* = 1128)	Kognitive Leistung, Anforderungsbewältigung, Affekt/Stimmung (ohne medizinische Indikation)	Rezeptpflichtige Stimulanzien (AMP, MPH, Antidementiva, Modafinil); illegale Stimulanzien (Kokain, AMP, METH); rezeptpflichtige affektmodulierende Substanzen (Antidepressiva, Betablocker, BZP); Cannabis	Rezeptpflichtige Stimulanzien: 2,2 %	Rezeptpflichtige Stimulanzien: 4,3 %
				Illegale Stimulanzien: 3,8 %	Illegale Stimulanzien: 10,2 %
				Rezeptpflichtige affektmodulierende Substanzen: 10,6 %	Rezeptpflichtige affektmodulierende Substanzen: 20,3 %
Compton et al. (2018, USA; [43])	Repräsentative Face-to-Face-Befragung Erwachsener (*n* = 102.000^a^)	Kognitive Leistung, Anforderungsbewältigung, Gewichtsreduktion (ohne medizinische Indikation)	Rezeptpflichtige Stimulanzien	Rezeptpflichtige Stimulanzien: 2,1 %	n. a.
Sattler et al. (2025, DE; [24])	Repräsentative Online-Befragung Erwachsener (*n* = 22.101)	Kognitive Leistung, Anforderungsbewältigung (ohne medizinische Indikation)	Legale Substanzen (koffeinhaltige Substanzen, Supplements, Hausmittel); rezeptpflichtige Substanzen (Stimulanzien, Antidementiva, Betablocker, Antidepressiva, Analgetika, Sedativa); Cannabis; illegale Substanzen (AMP, METH, Kokain)	Rezeptpflichtige Substanzen: 3,7 %	Rezeptpflichtige Substanzen: 5,5 %
				Cannabis: 1,6 %	Cannabis: 4,1 %
				Koffeinhaltige Getränke: 62,4 %	n. a.
				Koffeintabletten: 2,5 %	n. a.
				Supplements/Hausmittel: 31,4 %	n. a.
				Gesamt^b^: 69,9 %	n. a.
*Erwerbsarbeitende*					
Calvo et al. (2020, Spanien; [39])	Nichtrepräsentative Beobachtungsstudie körperlich tätiger Arbeiter:innen (*n* = 5623)	Schmerztherapie (ärztlich verordnet)	Nichtsteroidale Antirheumatika	41,6 %	n. a.
Dong et al. (2022, USA; [40])	Repräsentative Erhebung verschiedener Daten zu Bauarbeiter:innen (16+) (*n* = 9070^c^)	Schmerztherapie (ärztlich verordnet)	Rezeptpflichtige Analgetika (Opioide und Nichtopioide)	Rezeptpflichtige Opioide: 10,0 % Rezeptpflichtige Nichtopioide: 9,3 % Gesamt: 15,9 %	n. a.
Müller et al. (2020, DE; [21])	Computergestützte Interviews, repräsentativ für 4 Berufsgruppen (*n* = 4166)	Kognitive Leistung; Affekt/Stimmung (ohne medizinische Indikation)	Rezeptpflichtige Substanzen (Stimulanzien, Antidepressiva, Antidementiva, Sedativa, Opiate, u. a.); Cannabis; illegale Substanzen (Kokain, AMP)	Gesamt: 2,9 %	Gesamt: 8,4 %
Sattler und von dem Knesebeck (2022, DE; [25])	Repräsentative Online-Befragung Beschäftigter (*n* = 11.197)	Kognitive Leistung (ohne medizinische Indikation)	Rezeptpflichtige Substanzen (Stimulanzien, Antidepressiva, Antidementiva, Betablocker)	2,6 %	n. a.
*Studierende*					
Dietz et al. (2022, DE; [22])	3 nichtrepräsentative Online-Befragungen Studierender der JGU Mainz: vor (2019, *n* = 3984) und während der COVID-Pandemie (2020, *n* = 2796; 2021 *n* = 1232)	Kognitive Leistung, Anforderungsbewältigung (ohne medizinische Indikation)	Rezeptpflichtige Substanzen (Stimulanzien, Atomoxetin); Cannabis; illegale Substanzen (Ecstasy/MDMA, AMP, METH)	Gesamt: 10,4 % (2019); 11,3 % (2020); 8,0 % (2021)	n. a.
Heller et al. (2022, DE; [23])	Repräsentative Online-Befragung Studierender der JGU Mainz (*n* = 3984)	Kognitive Leistung, Anforderungsbewältigung (ohne medizinische Indikation)	Rezeptpflichtige Stimulanzien; illegale Stimulanzien (Ecstasy, AMP, METH, Cannabis)	Gesamt: 10,4 %	Gesamt: 15,1 %

^a^ Ungewichtete und gerundete Stichprobengröße. Die Prozentangabe der Prävalenzspalte ist gewichtet

^b^ Allgemeine Prävalenz pharmakologischen Neuroenhancements ohne Substanzdifferenzierung

^c^ Ungewichtete Stichprobengröße US-amerikanischer Bauarbeiter:innen. Die angegebenen Prävalenzwerte hingegen sind gewichtet und repräsentieren knapp 11,5 Mio. Arbeiter:innen jährlich, die zwischen 2011–2018 in der Bauindustrie angestellt waren

*AMP* amphetaminhaltige Präparate oder Substanzen; *MDMA* 3,4-Methylendioxy-N-methylamphetamin; *METH* Methamphetamin; *MPH* Methylphenidat; *n.* *a.* nicht angegeben oder nicht erhoben

Für den nichtmedizinischen Gebrauch verschreibungspflichtiger oder illegaler Substanzen werden in den von uns berücksichtigten Studien 12-Monats-Prävalenzwerte zwischen 2,1 % und 10,4 % berichtet. Eine bevölkerungsrepräsentative Studie aus den USA zeigt eine 12-Monats-Prävalenz von 2,1 % [43], während eine repräsentative Befragung aus Deutschland 12-Monats-Prävalenzen von 2,2 % bei rezeptpflichtigen und 3,8 % bei illegalen Stimulanzien angibt [29]. Studien, die Gesamtprävalenzen erfassen, berichten entsprechend deutlich höhere Werte: Sattler et al. (2025) berichten 12-Monats-Prävalenzen von über 3,7 % für rezeptpflichtige Stimulanzien und über 1,6 % für Cannabis. Die Gesamtprävalenz, die unter anderem koffeinhaltige Substanzen einschließt, liegt bei 69,9 % [24].

## Relevante Substanzgruppen

### Stimulanzien

Stimulanzien bilden eine Gruppe potenziell leistungssteigernder Mittel im Alltag und Arbeitsleben [10, 44]. Im Fokus dieser Betrachtung stehen verschreibungspflichtige Substanzen wie Methylphenidat, Amphetamin und seine Abkömmlinge sowie Modafinil, des Weiteren frei verfügbare Alltagsstimulanzien, insbesondere Koffein und Energy-Drinks. Verschreibungspflichtige Stimulanzien werden von gesunden Personen über die medizinischen Indikationen hinaus zur kognitiven Leistungssteigerung genutzt [10, 44, 45]. Nicht ärztlich überwachter Konsum ist mit kardiovaskulären Risiken sowie Stimmungs- und Schlafstörungen assoziiert [46]. Studien zeigen zudem, dass ein relevanter Anteil verordneter Medikamente gegen die Aufmerksamkeitsdefizit‑/Hyperaktivitätsstörung (ADHS) missbräuchlich genutzt oder weitergegeben wird, meist mit dem Ziel erhöhter Wachheit oder Konzentration, wobei kardiovaskuläre und psychiatrische Folgeerkrankungen auftreten können [47].

Koffeinhaltige Produkte wie Kaffee oder Energy-Drinks sind weitverbreitet und werden häufig zur Unterstützung von Wachheit, Aufmerksamkeit und Anforderungsbewältigung sowie aus sozialen Gründen genutzt [48, 49]*.* In moderaten Mengen kann Koffein Aufmerksamkeit und Konzentration kurzfristig verbessern, während hoher Konsum mit Abhängigkeit, Schlafstörungen, Angst und kardiovaskulären Effekten verbunden ist [49]. Energy-Drinks enthalten weitere stimulierende Substanzen und zeigen bei hohem Konsum akute Effekte auf Herzfrequenz und Blutdruck sowie Hinweise auf Herzrhythmusstörungen. Ihre Langzeitfolgen sind bislang unzureichend untersucht [50].

Insgesamt sind Nutzung und Erwartungen an Stimulanzien eng an Phasen erhöhter Belastung geknüpft, wobei subjektive Leistungsannahmen häufig über die empirisch belegten Effekte hinausgehen [5, 10]. Zentrale Risiken betreffen sowohl verschreibungspflichtige als auch frei verfügbare Stimulanzien und umfassen kardiovaskuläre und psychiatrische Folgeerkrankungen sowie Abhängigkeitsrisiken, weshalb in der Literatur vor einer weiteren Normalisierung des Konsums gewarnt wird [10, 47, 50].

### Sedativa und Hypnotika

Der Fokus der Literatur zu Mitteln zur Affekt‑, Stress- und Schlafregulation liegt auf Benzodiazepinen, sogenannten „Z-Substanzen“ (Zopiclon, Zolpidem), sowie Betablockern zur Schlafsteuerung. Übersichtsarbeiten zeigen, dass ihre Effekte meist nur kurzfristig anhalten, während Nebenwirkungen wie Tagesmüdigkeit, kognitive Beeinträchtigungen und Sturzrisiken persistieren [34, 36]. Zudem kann sich bereits nach wenigen Wochen eine Abhängigkeit entwickeln, weshalb sie für eine längerfristige Leistungs- oder Stressregulation ungeeignet sind [34].

Betablocker wie Propranolol werden zur Dämpfung peripherer Stressreaktionen eingesetzt. Zwar können sie vegetative Symptome von Leistungsangst mindern, jedoch zeigen sie keine konsistenten Effekte auf kognitive Leistungsfähigkeit oder subjektives Stresserleben. Nebenwirkungen wie Bradykardie, Hypotonie und Müdigkeit machen den Einsatz außerhalb klarer medizinischer Indikationen kritisch [51]. Zur Regulation von Schlaf und Wachheit wird Melatonin eingesetzt, etwa bei Schicht- und Nachtarbeit. Eine systematische Übersichtsarbeit zeigt für Melatonin jedoch nur geringe Effekte auf zentrale Schlafparameter wie die Schlafdauer [52]; für Hypnotika lassen sich nachhaltige Verbesserungen schichtarbeitsbezogener Schlafstörungen ebenfalls nicht konsistent belegen und die Langzeitrisiken bleiben insgesamt unzureichend geklärt [52].

Der Gebrauch von affekt-, stress- und schlafregulierenden Mitteln ist mit einem hohen Risiko für Toleranzentwicklung, Abhängigkeit und Residualeffekte verbunden. Es können neurokognitive Einschränkungen, verlangsamte Reaktionszeiten und sicherheitsrelevante Funktionseinbußen auftreten. Kurzfristige Entlastung geht deshalb häufig mit langfristigen gesundheitlichen und funktionalen Kosten einher [36].

### Analgetika und Antiphlogistika

Analgetika und antiphlogistische Medikamente werden im Alltag und im Arbeitsleben von Personen mit körperlich belastenden Tätigkeiten, wie etwa Bauarbeiter:innen, eingesetzt, um Schmerzen oder entzündliche Beschwerden zu lindern. Dieser Abschnitt fokussiert nicht die therapeutischen Anwendungen im engeren Sinne, sondern Situationen, in denen Medikamente funktional eingesetzt werden, um trotz körperlicher Beschwerden Arbeits- und Leistungsfähigkeit aufrechtzuerhalten, obwohl medizinisch Schonung, Regeneration oder eine vollständige Ausheilung der Grunderkrankung angezeigt wäre.

Eine 2022 veröffentlichte Studie verweist in diesem Zusammenhang auf das Risiko der Entwicklung von Opioidgebrauchsstörungen und betont die Notwendigkeit präventiver Strategien [40]. Vor diesem Hintergrund kann der wiederholte Einsatz solcher Substanzen über akute Indikationen hinaus als Hinweis auf eine funktional ausgerichtete Nutzung interpretiert werden, bei der die Aufrechterhaltung von Arbeitsfähigkeit im Vordergrund steht. Dies entspricht Konzepten wie dem „Präsentismus“ (s. oben; [37, 38]).

Ein aktuelles Scoping-Review aus dem Jahr 2023, das 65 Studien zu verschreibungspflichtigen Medikamenten bei Beschäftigten mit muskuloskelettalen Erkrankungen oder Verletzungen auswertete, zeigt, dass insbesondere Opioide, nichtsteroidale Antirheumatika (NSAR) und Muskelrelaxanzien zwar kurzfristige Schmerzlinderung bewirken, in der Mehrzahl der Untersuchungen jedoch mit ungünstigen arbeitsbezogenen Outcomes assoziiert sind, was sich entsprechend in längeren Fehlzeiten und eingeschränkter Arbeitsfähigkeit zeigt. Die Autorinnen und Autoren weisen zudem darauf hin, dass Opioide häufig über längere Zeiträume und in Konstellationen eingesetzt werden, in denen zunächst nichtopioide Analgetika empfohlen würden [41]. Diese Befunde spiegeln die Präsentismus-Logik wider. Der leistungsbezogene Gebrauch von schmerzlindernden Medikamenten fungiert damit als kurzfristige Bewältigungsstrategie, die strukturelle Überlastung und unzureichende Regenerationszeiten überdeckt und langfristig mit einem erhöhten Risiko chronischer Einschränkungen der Arbeitsfähigkeit einhergehen kann.

Die Risiken bei regelhaftem oder hochdosiertem Gebrauch sind substanzabhängig, insgesamt jedoch erheblich. Eine aktuelle Literaturübersicht zur kardiovaskulären Sicherheit von NSARs zeigt konsistent eine erhöhte Assoziation mit Ereignissen wie Myokardinfarkt, Schlaganfall und Hypertonie, wobei das Risiko bereits zu Therapiebeginn besteht und mit Dosis sowie Anwendungsdauer zunimmt. Dementsprechend wird eine zurückhaltende, zeitlich begrenzte Anwendung empfohlen [53]. Paracetamol kann bei hohen Dosierungen hepatotoxisch wirken, während opioidhaltige Analgetika bei längerfristigem Einsatz mit Toleranzentwicklung, Abhängigkeit und Überdosierungen einhergehen, was sich in ungünstigen arbeitsbezogenen Outcomes niederschlägt [41].

### Nahrungsergänzungsmittel

Nahrungsergänzungsmittel werden im Alltag und im Arbeitsleben zunehmend als „Nootropika“ (Substanzen, die die kognitive Leistungsfähigkeit, Stimmung oder Schlaf modulieren sollen) ohne ärztliche Indikation eingesetzt [54]. Dazu zählen unter anderem Ginkgo-biloba-Extrakte, Bacopa monnieri sowie Kombinationen aus L‑Theanin und Koffein. Der Gebrauch zielt weniger auf die Behandlung definierter Defizite ab als auf eine vermeintliche Optimierung oder Stabilisierung von Funktionsfähigkeit. Die herangezogene Literatur stützt sich vor allem auf neuere Übersichtsarbeiten zu Wirksamkeit, Sicherheit und regulatorischen Rahmenbedingungen [46, 54].

Viele dieser Nahrungsergänzungsmittel werden mit wissenschaftlich klingenden Marketing-Claims beworben, obwohl die Evidenz für einen relevanten Nutzen bei gesunden Personen begrenzt ist. Übersichtsarbeiten zeigen, dass sogenannte Smart Drugs ursprünglich zur Behandlung kognitiver Störungen entwickelt wurden, inzwischen jedoch auch von Gesunden breit genutzt werden, ohne dass belastbare Belege für eine nachhaltige Verbesserung exekutiver Funktionen vorliegen [46]. Gleichzeitig bestehen erhebliche Unsicherheiten hinsichtlich Wirksamkeit, Langzeitfolgen und Verbreitung.

Ein zentrales Risiko liegt in Qualitäts‑, Dosierungs- und Kombinationsproblemen. Studien zu unerlaubten Zusatzstoffen zeigen, dass ein erheblicher Anteil von Brain-Health-Produkten falsch deklariert oder mit nicht zugelassenen pharmakologisch wirksamen Substanzen versetzt ist [54]. Der Kombinationsgebrauch mehrerer Produkte (Stacking) erhöht das Risiko unerwünschter Wirkungen, da Dosierungen, Wechselwirkungen und tatsächliche Wirkstoffexposition häufig unklar sind. Insgesamt macht die Literatur deutlich, dass der leistungsbezogene Gebrauch von Nahrungsergänzungsmitteln bzw. „Nootropika“ häufig weder evidenzbasiert noch risikofrei ist. Unklare Nutzenannahmen, mangelhafte Produktqualität und unkontrollierter Kombinationsgebrauch stellen relevante gesundheitliche Risiken dar, insbesondere bei Nutzung ohne ärztliche Begleitung.

### Zusammenfassende Einordnung der Substanzgruppen

Die oben dargestellten Substanzgruppen lassen sich nach wiederkehrenden Motiven bzw. Funktionalitäten ordnen, wie der kurzfristigen Sicherung von Wachheit und Konzentration, der Regulation von Affekt, Anspannung und Schlaf sowie der Dämpfung körperlicher Beschwerden, um trotz Belastung funktionsfähig zu bleiben. Diese Perspektive entspricht einem instrumentellen Verständnis von Substanzgebrauch, bei dem Mittel gezielt eingesetzt werden, um situative Zustände und Handlungsfähigkeit zu steuern, ohne dass damit notwendigerweise eine nachhaltige Leistungssteigerung verbunden ist [55].

Stimulanzien werden häufig zur Leistungssteigerung eingesetzt [56], sind jedoch mit kardiovaskulären, psychiatrischen und schlafbezogenen Nebenwirkungen verbunden [10]. Affekt‑, Stress- und schlafregulierende Substanzen bergen Risiken wie Toleranzentwicklung, Abhängigkeit und Residualeffekte, Analgetika sind eng mit „Präsentismus“ und Symptommaskierung verknüpft. Bei Nahrungsergänzungsmitteln und Nootropika stehen oft unsichere Evidenzlagen Qualitäts- und Kombinationsrisiken gegenüber. Insgesamt zeigt sich, dass leistungsbezogener Substanzgebrauch weniger durch einzelne Stoffe als durch wiederkehrende Funktionslogiken geprägt ist, bei denen kurzfristige Entlastung häufig mit langfristigen gesundheitlichen und funktionalen Kosten erkauft wird.

## Diskussion

Die Evidenz zu Nutzen und Risiken leistungsbezogenen Substanzgebrauchs im Alltag und im Arbeitsleben deutet substanzgruppenabhängig auf heterogene [21, 24] und begrenzte Effekte hin [6, 45, 57]. Zwar sind kurzfristige Effekte auf Wachheit, Aufmerksamkeit oder subjektive Entlastung in Teilen belegt, sie betreffen jedoch meist Teilleistungen und gehen mit relevanten Kostenabwägungen einher. Kurzfristige Leistungsstabilisierung kann zulasten von Schlaf, Erholung, emotionaler Stabilität, Urteilsqualität und langfristiger Gesundheit erfolgen. Subjektive Erwartungen an Leistungsgewinne übersteigen dabei häufig die empirisch belegten Effekte, sodass leistungsbezogener Konsum weniger als nachhaltige Optimierung denn als situatives Instrument zur Überbrückung von Anforderungen erscheint.

Ein zentraler Risikotreiber ist der Kombinationsgebrauch. Mischkonsum entsteht häufig funktional, etwa durch den Wechsel zwischen stimulierenden und sedierenden Substanzen oder durch parallele Nutzung von Analgetika und Stimulanzien. Dadurch erhöhen sich die Risiken für Wechselwirkungen, Dosiseskalation und unbeabsichtigte Nebenwirkungen, insbesondere bei frei erhältlichen oder falsch deklarierten Produkten. Für Alltag und Arbeitswelt sind zudem Residualeffekte sicherheitsrelevant: Sedierung, verlangsamte Reaktionszeiten oder eingeschränkte exekutive Funktionen können insbesondere in verantwortungskritischen Tätigkeiten erhebliche Risiken darstellen. Symptommaskierung trägt darüber hinaus dazu bei, Warnsignale zu übergehen und Belastungsgrenzen systematisch zu verschieben.

## Fazit

Insgesamt zeigt sich, dass leistungsbezogener Substanzgebrauch kein Randphänomen ist und eng mit Arbeitsanforderungen, Zeitdruck, Mehrfachbelastungen und begrenzten Erholungsressourcen verknüpft ist. Aus Public-Health- und arbeitsmedizinischer Perspektive ergeben sich daraus klare Konsequenzen. Leistungsbezogener Substanzgebrauch sollte als Indikator für belastende Arbeits- und Lebensbedingungen verstanden und frühzeitig in Prävention, Beratung und Versorgung aufgegriffen werden, ohne ihn auf individuelles Fehlverhalten zu verkürzen. Erforderlich sind realistische, nicht moralisierende Risikoinformationen, die insbesondere Kombinationsgebrauch und sicherheitsrelevante Effekte adressieren. Darüber hinaus müssen strukturelle Rahmenbedingungen wie Arbeitsverdichtung, Schichtsysteme, Erreichbarkeitsnormen und Präsentismus-Druck stärker berücksichtigt werden. Wirksame Prävention erfordert integrierte Ansätze, die individuelle Unterstützung, medizinische Versorgung und gesundheitsförderliche Arbeitsgestaltung verbinden und leistungsbezogenen Substanzgebrauch als Frühwarnsignal für eine unzureichende Passung zwischen Anforderungen und Ressourcen nutzen.

## Data Availability

Alle dieser Arbeit zugrunde liegenden Daten sind in diesem Artikel enthalten.

## Footnotes

1 https://www.uni-bielefeld.de/fakultaeten/soziologie/forschung/projekte/enhance/; letzter Zugriff am 20.03.2026.
